# Molecular Characterization of the First Bovine Herpesvirus 4 (BoHV-4) Strains Isolated from *In Vitro* Bovine Embryos production in Argentina

**DOI:** 10.1371/journal.pone.0132212

**Published:** 2015-07-15

**Authors:** Erika González Altamiranda, Julieta M. Manrique, Sandra E. Pérez, Glenda L. Ríos, Anselmo C. Odeón, María R. Leunda, Leandro R. Jones, Andrea Verna

**Affiliations:** 1 Laboratorio de Virología, Departamento de Producción Animal, Instituto Nacional de Tecnología Agropecuaria (INTA), Balcarce, Buenos Aires, Argentina; 2 Laboratorio de Virología y Genética Molecular, Facultad de Ciencias Naturales Sede Trelew, Universidad Nacional de la Patagonia San Juan Bosco, Chubut, Argentina; 3 Facultad de Ciencias Veterinarias, Universidad Nacional del Centro de la Provincia de Buenos Aires (UNCPBA), Sede Tandil, Buenos Aires, Argentina; 4 Laboratorio de Producción de Embriones, Departamento de Producción Animal, Instituto Nacional de Tecnología Agropecuaria (INTA), Balcarce, Buenos Aires, Argentina; 5 Consejo Nacional de Investigaciones Científicas y Técnicas (CONICET), Buenos Aires, Argentina; Utah State University, UNITED STATES

## Abstract

Bovine herpesvirus 4 (BoHV-4) is increasingly considered as responsible for various problems of the reproductive tract. The virus infects mainly blood mononuclear cells and displays specific tropism for vascular endothelia, reproductive and fetal tissues. Epidemiological studies suggest its impact on reproductive performance, and its presence in various sites in the reproductive tract highlights its potential transmission in transfer-stage embryos. This work describes the biological and genetic characterization of BoHV-4 strains isolated from an *in vitro* bovine embryo production system. BoHV-4 strains were isolated in 2011 and 2013 from granulosa cells and bovine oocytes from ovary batches collected at a local abattoir, used as “starting material” for *in vitro* production of bovine embryos. Compatible BoHV-4-CPE was observed in the co-culture of granulosa cells and oocytes with MDBK cells. The identity of the isolates was confirmed by PCR assays targeting three ORFs of the viral genome. The phylogenetic analyses of the strains suggest that they were evolutionary unlinked. Therefore it is possible that BoHV-4 ovary infections occurred regularly along the evolution of the virus, at least in Argentina, which can have implications in the systems of *in vitro* embryo production. Thus, although BoHV-4 does not appear to be a frequent risk factor for *in vitro* embryo production, data are still limited. This study reveals the potential of BoHV-4 transmission via embryo transfer. Moreover, the high variability among the BoHV-4 strains isolated from aborted cows in Argentina highlights the importance of further research on the role of this virus as an agent with the potential to cause reproductive disease in cattle. The genetic characterization of the isolated strains provides data to better understand the pathogenesis of BoHV-4 infections. Furthermore, it will lead to fundamental insights into the molecular aspects of the virus and the means by which these strains circulate in the herds.

## Introduction

Bovine herpesvirus 4 (BoHV-4) is a member of the *Herpesviridae* family, *Gammaherpesvirinae* subfamily, *Rhadinovirus* genus. BoHV-4 was isolated for the first time in 1963 from animals with respiratory and ocular signs in Europe [1]. Afterwards, virus isolates were classified into two groups by restriction endonuclease analysis [2–3]: Movar 33/63-like strains, isolated from Europe, and DN 599-like strains, isolated from North America [2–4]. In 2007, BoHV-4 was isolated in Argentina from vaginal secretions of aborted cows [5] and later from buffy coat fractions in association with bovine viral diarrhea virus [6]. More recently, the virus was isolated from bovine semen from an artificial insemination center [7].

Abortions associated with BoHV-4 are usually sporadic. Currently, the exact role of this virus in cattle diseases is unknown. However, several research groups [8–11] are studying its participation in the infection of the genital tract. Most reports of BoHV-4 infection in different countries associate BoHV-4 with the development of post-partum and chronic metritis, either alone or in combination with other pathogens [12–13]. Data on the relationship between BoHV-4 and infertility are limited [14]. However, a recent study has shown a positive correlation between infertility and BoHV-4 infection [15]. The presumptive implication of BoHV-4 in reproductive disorders is based on epidemiological observations, especially a higher seroprevalence in bovine females affected by metritis, abortion and/or infertility than in healthy bovine females [15–17].

Recently, Gur and Dogan [15] investigated the role of BoHV-4 infections in repeat breeding cows by serology and they found that BoHV-4 seropositivity is associated with increased risk of infertility: 69% of repeat breeder females (3–14 artificial inseminations) presented anti-BoHV-4 antibodies whereas only 44% of the cows that had received two inseminations or less were seropositive. However, such correlation was based only on epidemiological observations. In another recent study, the virus was isolated from 87% of the uteri from infertile cows obtained at slaughterhouses [18], indicating a high prevalence of BoHV-4 in reproductive tissues. These ‘materials of animal origin’ represent an important source of contamination when used in production and transfer of bovine embryos.

The virus has also been identified in aborted fetuses, mainly in spleen lymphocytes and monocytes and, occasionally from hepatic Kupffer cells and renal tubules [16–20]. It has also been evidenced in placental epithelium, where it replicates (especially within intraplacental inflammatory infiltrates) without causing any specific histological lesions. Because the virus has been isolated from various sites of the reproductive tract, it can be potentially transmitted by transfer-stage embryos [20]. Recent studies have shown other possible sources of infection such as spermatozoa, semen leukocyte fractions and colostral cells in which the viral genome was detected by PCR, indicating that reproductive cells might be a target for the virus [21].

The study of bovine herpesviruses in *in vitro*-produced embryos has been focused mainly on bovine herpesvirus-1 (BoHV-1) [22] and more recently, on bovine herpesvirus 5 (BoHV-5) [23–24]. However, there is little information on the infections by BoHV-4 in *in vitro* systems of embryo production. As detailed below, we successfully isolated BoHV-4 from granulosa cells and oocytes in 2011 and 2013, respectively. In the following years the virus was frequently isolated from samples of aborted cows, and hence we here describe a detailed characterization of the 2011 and 2013 strains.

## Materials and Methods

### Case History and Virological Findings

BoHV-4 strains were isolated in 2011 and 2013 from bovine granulosa cells and oocytes from batches of ovaries collected at a local abattoir located in Balcarce, Argentina (37° 50' 47” S, 58° 15' 19” W). Bovine ovaries were collected from a local slaughterhouse and transported in a thermic container to the Laboratory of *in vitro* Embryo Production (INTA), which is regulated by the principles established by the International Embryo Transfer (IETS) to assure quality and sanitary standards (that is, free of pathogens). Groups of 50 cumulus-oocyte complexes (COCs) corresponding to grade 1 and 2, were selected under a stereomicroscope and placed in four-well dishes (Nunc) in 400 μL of standard maturation medium (M199 plus 0.1 mg/mL L-glutamine and 2.2 mg/mL NaHCO_3_ was supplemented with 0.01 IU/mL rh-FSH [Gonal F-75, Serone, UK] and 10% estrous cow serum). After maturation, COCs were completely denuded of cumulus cells by pipetting in M199-HEPES containing 300 IU/mL hyaluronidase (H3506) for 2 min. Cumulus-free oocytes were washed five times in medium drops.

The material obtained from granulosa cells (30 ul), oocytes (n = 10) and washed medium drops (30ul) were co-cultured on confluent Madin-Darby bovine kidney (MDBK) cells grown in 96-well tissue culture plates. MDBK cells were incubated in minimum essential medium (MEM) supplemented with 10% fetal bovine serum. MDBK cells were provide by the ABAC (Argentinean Cell Bank, http://www.abac.org.ar/). They were BoHV-4-free, and certified as free of contaminating bacteria, mycoplasma and adventitious viruses.

Samples were subjected to three blind passages (48 h apart) at 37°C, 5% CO_2_ and observed daily for the presence of cytopathic effect (CPE). MDBK cells were mock-infected in parallel to provide a negative control. In addition, control procedures included routine PCR assessments of wash and culture media used for all the experiments.

### Detection of Viral DNA by PCR

In all cell cultures DNA was purified from infected cells using the DNeasy Blood & Tissue Kit (Cat. 69504, Qiagen), according to the manufacturer's instructions. DNA concentration was determined by spectrophotometry at an absorbance of 260 nm. The presence of BoHV-4 DNA was evaluated by PCR assays targeting three ORFs of the viral genome: ORF8 (encoding glycoprotein B) [25], ORF22 (encoding glycoprotein H) [26] and ORF47 (encoding glycoprotein L) [27]. The BoHV-4 thymidine kinase (TK) gene was evaluated by a nested-PCR assay modified by [5]. The primers used for the amplifications are described in Table 1. DNA from mock-infected MDBK cells was used as negative control. The specificity of the reactions was evaluated by using DNA from reference strains of BoHV-1 and BoHV-5 as PCR template. The amplified products were separated by electrophoresis in a 2% agarose gel, and DNA bands were visualized by SYBR Safe DNA gel stain (Invitrogen).

**Table 1:** Primers used in this study.

Gene	GeneBank accession no.	Orientation	Primer	Fragment expected (bp)
ORF22	AF318573	Forward	5’-CCGGGTGAAACAAGTTCCTG-3’	563
		Reverse	5’-GTCAGAGAACATATGATAACATC-3’	
ORF47	AF318573	Forward	5’-CAGGCAAAAAGTGGCTTCTC-3’	138
		Reverse	5’-TTGAGGGCCTGGATATTGTC-3’	
ORF8	Wellenberg et al., 2001	Forward	5’-CCCTTCTTTACCACCACCTACA-3’	615
		Reverse	5’-TGCCATAGCAGAGAAACAATGA-3’	
Orf3	Verna et al., 2012	Foward	5’-GTTGGGCGTCCTGTATGGTAGC-3’	576
		Reverse	5’-TGTATGCCCAAAACTTATAATATGACCAG-3’	
Orf3	Verna et al., 2012	Foward	5’-TTGATAGTGCGTTGTTGGGATGTGGT-3’	216
		Reverse	5’-CACTGCCCGGTGGGAAATAGCA-3’	

* The pairs of primers used in this study were designed based on the sequences available in GenBank.

### Sequencing and Evolutionary Analyses

The PCR amplicons obtained as described above were subjected to Sanger sequencing using PCR primers. Furthermore, the gB gene from 11 Genotype 1 strains (isolates 07_568, 08_433, 09_467, 09_759, 08_209, 10_154, 09_465, 08_362, DN_599, 08_476 and 08_263), two Genotype 2 strains (09_227, 07_435) and one strain (08_404) of uncertain parentage were also amplified and sequenced for their use in evolutionary analyses. The sequences described here were deposited in GenBank under accession numbers. The herein reported nucleotide sequences have been assigned GenBank accession numbers KP209014- KP209031.

For the evolutionary analyses, the TK sequences described here were combined with TK sequences of isolates 07_568, 08_433, 09_467, 09_759, 08_209, 10_154, 09_465, 08_362, DN_599, 08_476 and 08_263, 09_227, 07_435 and 08_404, described before [6]. We also determined the gB sequences of these isolates. After alignment and trimming of terminal alignment columns containing missing data, our dataset contained 435 positions of the gB gene and 212 from TK, of which 43 were informative. Sequence alignments were generated by the MAFFT multiple sequence alignment program, with default *op* and *ep*, and iterative refinement and weighted sum-of-pairs and consistency scores obtained from local alignments [28]. Numbers of informative sites were determined using the Phyloch package (http://www.christophheibl.de/Rpackages). Evolutionary models for the alignments obtained were selected with the MrAIC.pl. program distributed by the author [29]. These last analyses favored a JC69 model for both datasets.

Maximum Likelihood phylogenetic trees were inferred with the FastTree 2 program [30]. FastTree implements a Maximum Likelihood method of phylogenetic tree inference. The Maximum Likelihood approach evaluates a hypothesis in terms of the probability that a model and a hypothesized history would give rise to the observed sequences, which is to say the probability of the sequence alignment given the tree. It is assumed that the history (tree) associated with the higher data probability is preferred in comparison with a tree with a lower probability [31]. FastTree automatically scale search intensities according to dataset sizes by using up to 4xlog2(N) rounds of minimum-evolution nearest neighbour interchange, 2 rounds of subtree pruning and regraft moves and up to 2xlog(N) rounds of maximum-likelihood nearest neighbour interchanges, where N is the number of unique sequences in the dataset [30]. For the bootstrapped analyses, 100 resampled datasets were generated with the *Seqboot* component of the *Phylip* package [32], and subjected to FastTree 2 and the *CompareToBootstrap*.*pl* PERL script provided with the program.

Bayesian coalescent analyses were performed with *BEAST* [33]. BEAST is a program that implements a Bayesian framework for testing evolutionary hypotheses without conditioning on a single tree topology. This is done exploring the tree and parameter space by a Markov Chain Monte Carlo approach. Thus phylogenies and associated parameters are weighted in proportion to their posterior probabilities. A detailed description of the program and concepts implemented in the program can be found in [34]. The BEAST results were analyzed using Tracer v1.5 (available from: http://tree.bio.ed.ac.uk/software/tracer/). We performed two independent runs of 5E7 generations, which were inspected for convergence, likelihood; prior and posterior yielded an Effective Sample Size (ESS) greater than 200, and visually to check if there were any trends in the trace of each parameter. The runs were merged with LogCombiner (distributed with BEAST). The gB and TK datasets were combined into a single Bayesian coalescent analysis. However, the parameters of the corresponding site models, clocks and trees were unlinked, as preliminary phylogenetic analyses supported different evolutionary histories for the genes studied (please see also the following sections). Log-normal, relaxed molecular clocks were set, with uniform priors in the range of mutational rates known for herpesviruses and other double-stranded DNA viruses [35]. Although the isolation dates of the strains studied here are known, we considered the time interval encompassed by these dates to be too narrow in comparison with the evolutionary time scales of herpesviruses; *i*.*e*. codivergence with host species [36]. Thus, tip dates were not used to estimate the evolutionary rates and dates, but let them follow the prior. Convergence and adequate EES (Target 1000) were assessed with Tracer (available from http://beast.bio.ed.ac.uk/). The posterior sample of trees were summarized with TreeAnnotator [33].

## Results

### Characterization of Isolates

**Fig 1:**
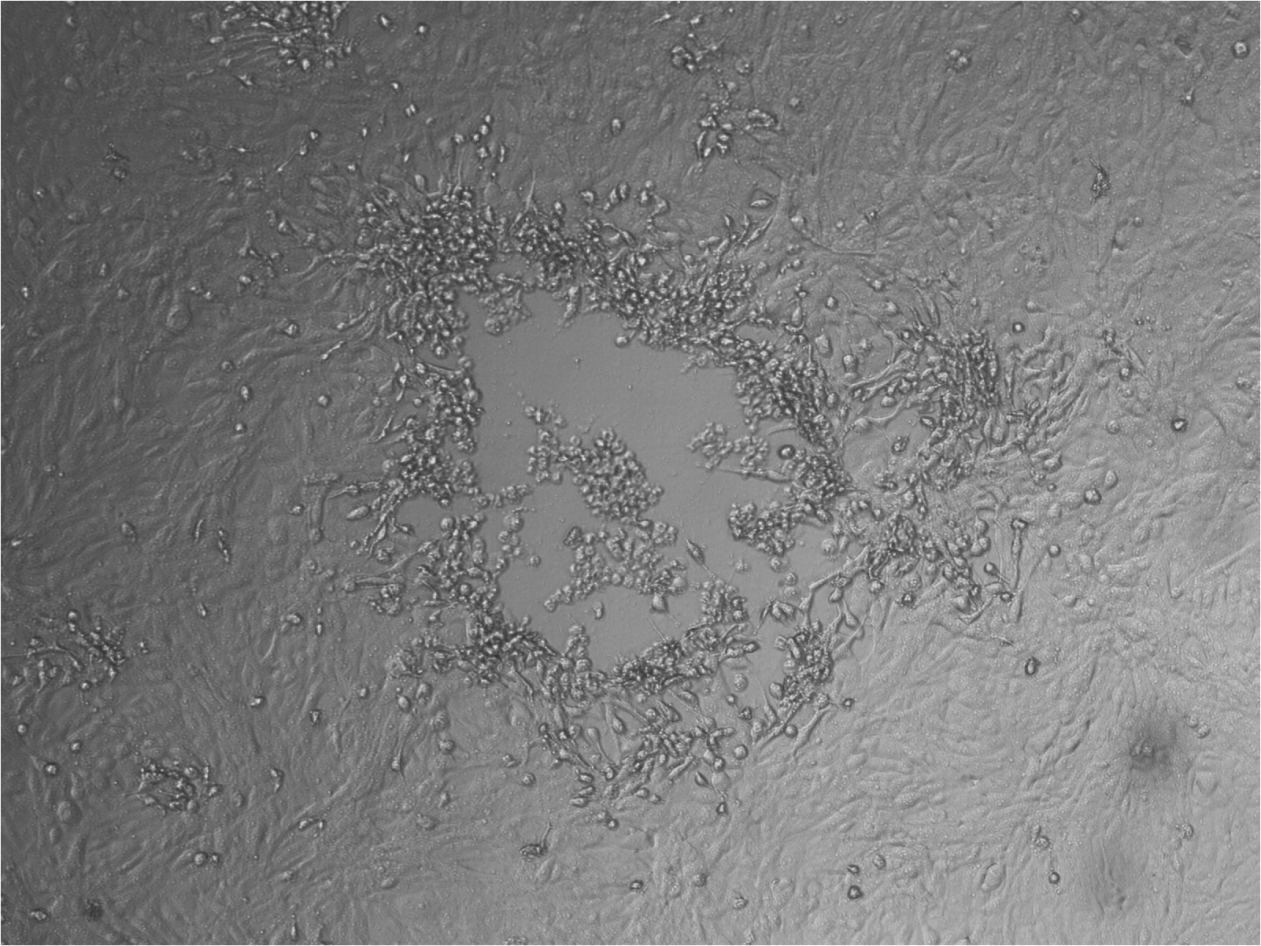
Cytopathic effect induced by BoHV-4 strains from bovine ovary batches on MDBK cells. Cytopathic effect associated with BoHV-4 infections. Light microscope (10X).

After oocyte maturation, granulosa cells were removed from oocytes and both samples were co-cultured with MDBK cells. After three blind passages, CPE resembling that of BoHV-4 was observed in the co-culture of granulosa cells and oocytes with MDBK cells but not in control ones (Fig 1). PCR and IF (FITC anti-BoHV-4 monoclonal antibody, Bio-X Diagnostics S.P.R.L.U) were used to confirm the presence of the virus in the inoculated cells.

DNA was obtained from both samples and subjected to the PCR reactions described in Material and Methods (Table 1). A specific band for the amplified fragments was obtained from the DNA of oocytes and granulosa cells. As expected, BoHV-4 DNA was not detected in non-infected MDBK cells.

### Evolutionary Analyses

**Fig 2:**
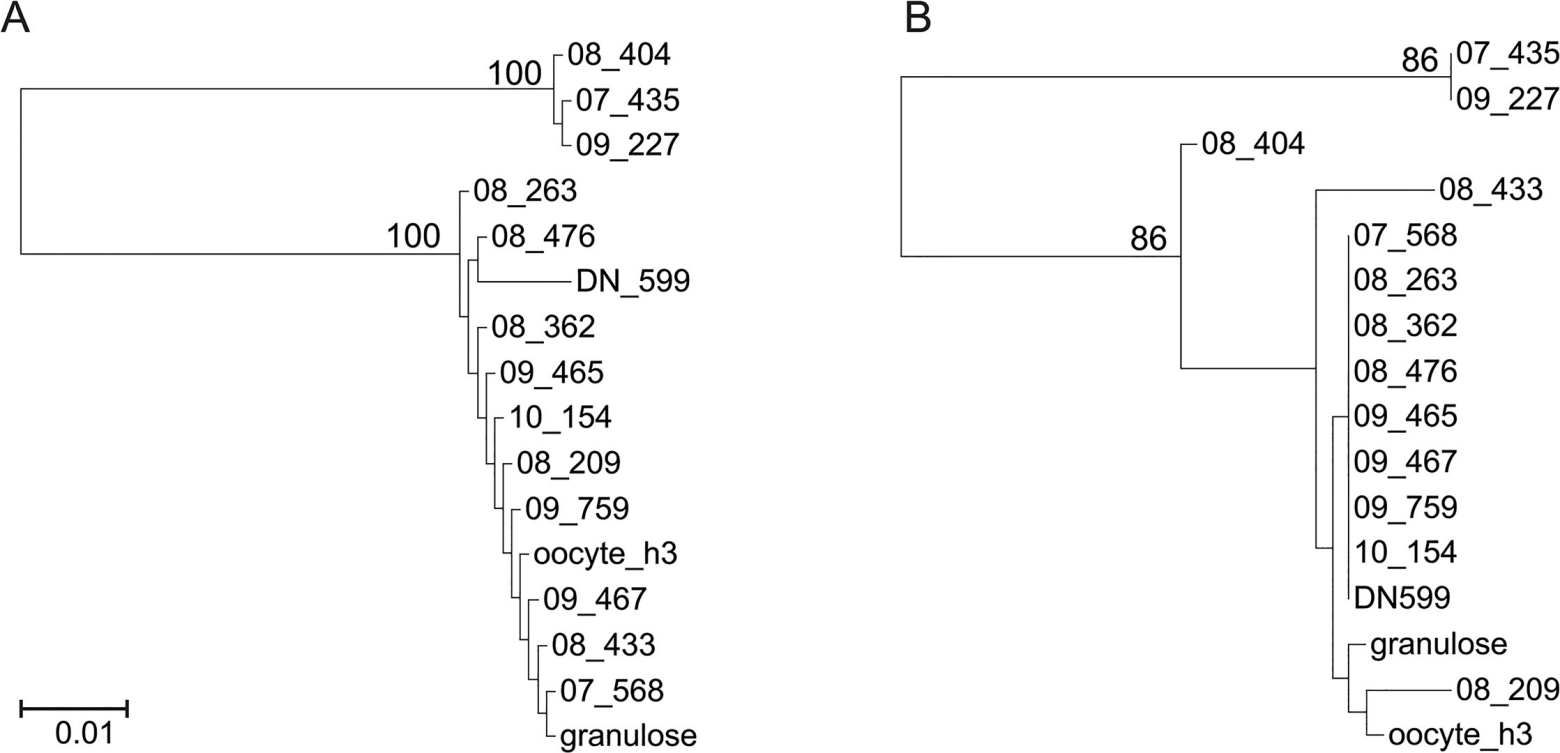
Phylogenetic analysis of the BoHV-4 strains studied here. (A) Obtained from glycoprotein B gene sequence. (B) Obtained from thymidine kinase gene sequence. Branch lengths are proportional to genetic distances (the scale bar units are substitutions per aligned position). Numbers close to nodes correspond to bootstrap supports (n = 100); non-significant bootstrap supports are not shown. The trees were midpoint rooted.

**Fig 3:**
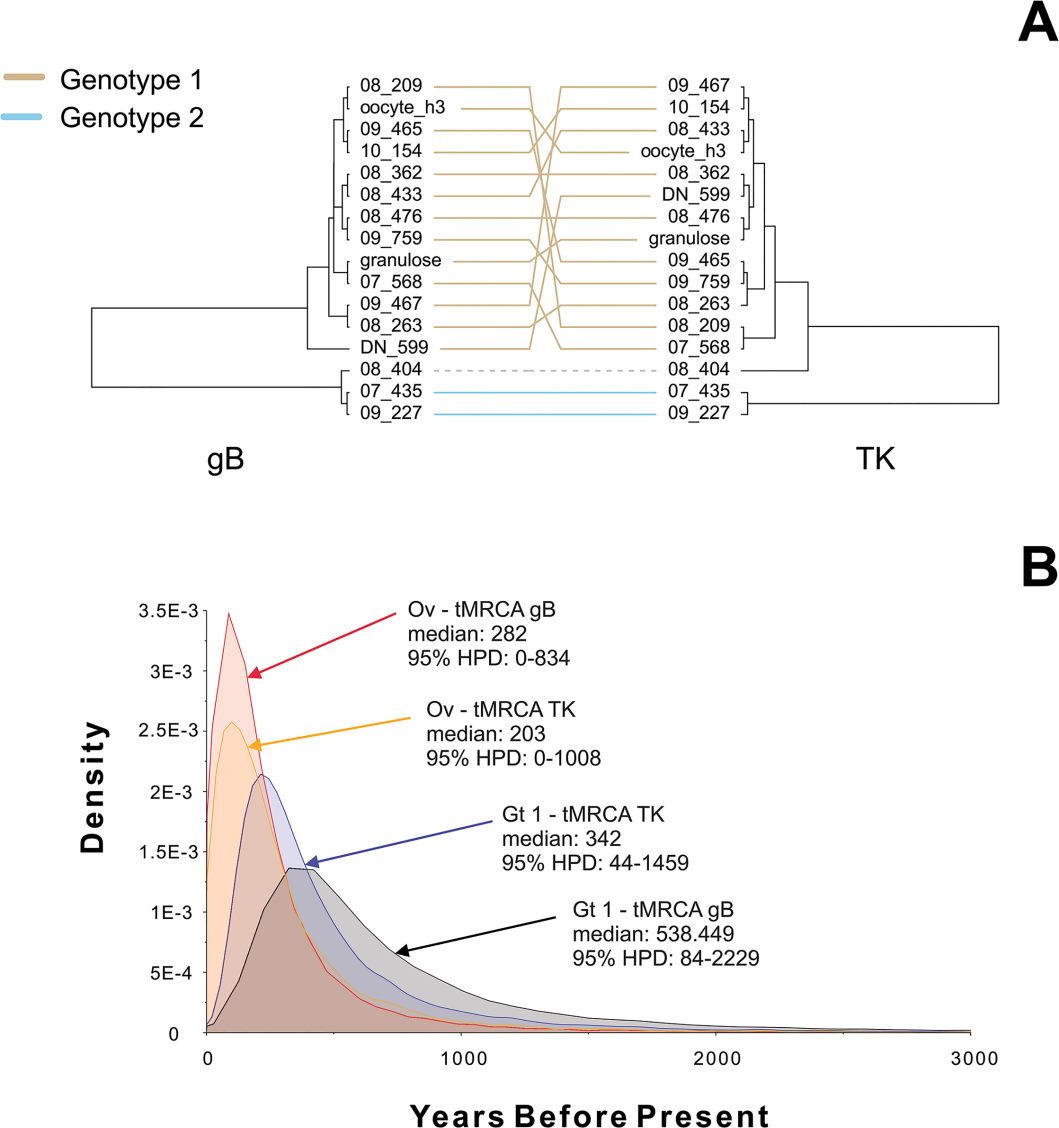
Bayesian Evidence for the BoHV-4 sequences studied here. (A) Mirrored maximum clade credibility trees. (B) Times to most recent common ancestor (tMRCA) posterior densities, medians, and 95% HPD intervals of the studied strains are shown. Branch lengths are proportional to median node heights. The links connecting terminal nodes are colored according to which genotype the corresponding strains belong to (*tan* Genotype 1; *light blue* Genotype 2). The link connecting terminals corresponding to strain 08_404 are dashed and gray colored to indicate uncertainty in genotype assignment. *Ov* strains isolated from ovaries (*granulosa cells* and *oocyte_h3*); *Gt1* Genotype 1; *gB* glycoprotein B; *TK* thymidine kinase.

Phylogenetic comparisons with previously described BoHV-4 sequences indicated that the strains obtained from the bovine ovaries collected at slaughterhouse belonged to Genotype 1. However, there were conspicuous differences in the branch length and topology between the trees obtained from the genomic regions studied. For example, in the gB tree, the strain 08_263 was much closer to Genotype 1 viruses than in the TK phylogeny (Fig 2). Likewise, in the TK tree, a relatively large branch connected strain 08_433 to the base node of Genotype 1, whereas it displayed a shorter branch emerging from inside Genotype 1 clade in the gB tree (Fig 2). Fig 3A depicts in detail the topological incongruence observed for both genes using mirrored trees. The observed level of incongruence is consistent with the evolutionary scenarios observed for other herpesviruses [37]. As for the ovary sequences, they belonged to Genotype 1. However, they were unconnected to each other in both the TK and gB trees (Figs 2 and 3).

The Bayesian analyses revealed a mean time to most recent common ancestor (tMRCA) of 808.85 (95% Highest Posterior Density (*HPD*) interval 9.49–2234.49) and 279.50 (95% HPD interval 0.18–941.01) years for the gB and TK Genotype 1 sequences, respectively. The tMRCAs of the ovary sequences were 111.29 (95% HPD interval 0.00–393.08) and 154.54 (95% HPD 0.00–579.26) years for the gB and TK genes, respectively. The posterior densities for these data are provided in Fig 3B.

## Discussion

Many studies have shown the effects of BoHV-4 on the reproductive tract. Nevertheless, there are no reports on the role of BoHV-4 in bovine infertility. In the present study, two BoHV-4 strains were isolated from granulosa cells and oocytes from ovaries obtained at slaughterhouse. The authenticity of the isolates was confirmed by PCR and sequencing of the TK locus as a genetic marker for the BoHV-4 genome, the 3' end of ORF1 (homologous to the EBV BVRF1 gene), ORF2 (homologous to the EBV BXRF1 gene), ORF3 (TK gene) and ORF22 (gH gene). This is the first report on the isolation and genomic characterization of BoHV-4 strains from ovaries obtained at slaughterhouse, which are routinely used as source of material for techniques of assisted reproduction in cattle.

The phylogenetic analyses of the strains isolated from bovine ovaries suggest that these strains were evolutionary unlinked. Therefore, it is possible that BoHV-4 ovary infections occurred regularly along the course of the evolution of the virus, at least in our country, which can have implications in the systems of *in vitro* embryo production, as discussed below. This phenomenon was also supported by the Bayesian analyses (Fig 3), which showed that the viruses isolated from the ovaries belong to lineages that have been circulating for long in our country, as can be inferred from the corresponding tMRCAs, which dates back to a period comprised among 0–834 (gB gene) or 0–1008 (TK gene) years before the isolation date. Both time periods widely overlap with the MRCA of genotype 1, strongly suggesting that all the strains from this genotype might be capable of producing ovary infections or at least be transmitted by ovary tissues. Furthermore, it should be noticed that cattle was introduced in Argentina around 400–500 years ago. Thus, based in the Bayesian tMRCAs, it is hypothesized that the lineages to which the ovary strains belongs to were introduced at that time. These data provide insight on the historical co-occurrence of ovary and "regular" infections. Both the chronograms obtained and the broad overlapping of the posterior density estimates of the tMRCAs are consistent with the recurrent infections of reproductive organs along the evolution of BoHV-4 in our country. These results suggest that BoHV-4 strains isolated from the reproductive tract belong to a viral lineage that has circulated for a relatively long period of time, contrary to the idea that the isolates correspond to a new viral variant with a specific tropism. Furthermore, these results support the concept that BoHV-4-related reproductive problems could be frequent and that, therefore, the virus represents a risk for the embryo production industry. In the case of BoHV-4, the information on this aspect is scarce, mainly because direct association between virus infection and disease has not been determined yet [35]. However, some studies have attempted to understand the interaction between the virus and *in vitro*-produced bovine embryos. Stringfellow et al. [20] demonstrated that BoHV-4 attaches to the intact zona pellucida of bovine embryos and that only trypsin treatment is able to remove the virus. On the other hand, in a preliminary study by using a green recombinant BoHV-4, it has been shown that the virus has low ability to infect *in vitro*-produced bovine embryos, which depends on the absence of zona pellucida, the amount of virus present and the stage of embryonic development [38]. In spite of these preliminary studies based on bovine embryos, there are no reports on the outcome of the interaction between BoHV-4 and gametes.

In the present work, viral isolation from oocytes with intact zona pellucida after several consecutive washes was consistent with the results obtained by [20]. This result has important implications about the effects of early BoHV-4 infections on vertical transmission. Recent studies have postulated that the virus is also able to cross the placental barrier and intensively replicate within the fetus [39]. However, it should be addressed that oocytes in this study were obtained from ovaries at slaughterhouse, which implies a possible natural infection of the host. It has been suggested that by placental cell destruction, induction of placental inflammation or alteration in the immune balance associated with pregnancy, the virus might alter placental function, leading eventually to delayed abortion [40].

Thus, although BoHV-4 does not appear to be a frequent risk factor for *in vit*ro embryo production, data are still limited. However, this study reveals that the potential of BoHV-4 transmission via embryo transfer exists. Moreover, high variability among the BoHV-4 strains isolated from aborted cows in Argentina highlights the importance of further research on the role of this virus as an infectious agent able to cause reproductive disease in cattle [6]. The genetic characterization of the isolated strains can therefore provide data to better understand the pathogenesis of BoHV-4 infections. Furthermore, strain characterization will lead to fundamental insights into molecular aspects of the virus and the means by which these strains circulate in the herds.
